# CT-guided lung biopsy service with conservative management of pneumothorax

**DOI:** 10.1016/j.fhj.2025.100471

**Published:** 2025-09-19

**Authors:** Syed Hasan Mustafa Rizvi, Avik Banerjee, Georgios Tsaknis, Muhammad Naeem, Syed Mehdi, Samantha Rawson, Raja Reddy

**Affiliations:** Respiratory and Pleural Department, Kettering General Hospital, Kettering, United Kingdom

**Keywords:** CT-guided lung biopsy, Pneumothorax, Ambulatory care, Outpatient management, Healthcare efficiency

## Abstract

**Background:**

CT-guided lung biopsy (CTGB) is a critical diagnostic tool for pulmonary lesions but is associated with a significant risk of pneumothorax, requiring inpatient observation in some cases. Limited hospital bed availability within the NHS constrains procedural capacity and prolongs waiting times. This study evaluates the safety and efficiency of an outpatient CTGB service integrated with an ambulatory pneumothorax management pathway.

**Methods:**

A retrospective analysis of 213 patients scheduled for CTGB between March 2021 and June 2023 was conducted. Patients developing pneumothorax were stratified for outpatient management based on haemodynamic stability, symptom severity and home support availability. Follow-up included clinical assessments and serial chest X-rays at 24–48 h, 1 week, 3 weeks and 7 weeks post-procedure.

**Results:**

Of 207 patients who underwent CTGB, 42 (20.2%) developed pneumothorax, with 39 meeting ambulatory management criteria. Thirty-eight (90.4%) were successfully managed without immediate intervention, and four (9.5%) required later intervention, including chest drain insertion or aspiration. The implementation of the conservative management of pneumothorax increased procedural capacity from two to three biopsies per session, reducing waiting times without compromising patient safety.

**Conclusion:**

Ambulatory management of post-CTGB pneumothorax is safe and effective, reducing inpatient admissions and optimising hospital resources. The structured follow-up pathway ensured patient stability, with minimal need for escalation of care. Our findings support broader implementation of this model to improve healthcare efficiency while maintaining high standards of patient care.

## Highlights

• Most patients who develop iatrogenic pneumothorax following CTGB can be managed conservatively via ambulatory care.
• CTGB of lung can be done without reserving an inpatient recovery bed for patient undergoing the procedure.
• Most patients with small pneumothorax can be discharged after they are observed for 1 h and had repeat CXR.

## Introduction

Lung biopsy is a widely used diagnostic procedure that plays a critical role in diagnosing lung cancer and other pulmonary disorders.^1^ The procedure typically involves obtaining a tissue sample using a percutaneous needle inserted through the chest wall, guided by imaging techniques such as computed tomography (CT) or fluoroscopy. Among these methods, CT-guided lung biopsy (CTGB) has become the preferred approach due to its superior precision in targeting lesions and its comparatively lower risk of complications.^2^

However, CTGB is not without risks. Pneumothorax remains the most common complication.^3^ It canoccur in up to 61% of cases, with approximately 3.3–15% requiring chest drainage.^4^ Given these potential complications, most CTGB procedures are performed on a day-case basis, often necessitating a short period of inpatient observation of vital signs and access to a day-case recovery bed for clinical observation.^2^ This requirement presents a significant challenge within the NHS, where bed availability is a persistent issue.^5^ Ewbank *et al* (2021) highlighted that the UK has fewer acute hospital beds per capita compared with many comparable healthcare systems.^6^ Limited day-case bed availability for post-procedural observation or pneumothorax management can constrain the number of CTGBs that can be performed in a session, leading to procedural delays and extended waiting times.

To address these challenges, we implemented a novel CT-guided lung biopsy service integrated with our pre-existing conservative pneumothorax management pathway. This initiative aimed to safely manage iatrogenic pneumothorax on an ambulatory basis, reducing the need for booked recovery beds and optimising procedural capacity. By shortening the post-procedure observation period and eliminating unnecessary inpatient admissions, we sought to improve efficiency while maintaining high standards of patient safety and care.

**AIM:** The aim of the study is to evaluate safety and efficacy of a CTGB service integrated with a conservative pneumothorax management pathway.

## Methods

### Patient selection and pre-procedural assessment

Patients with suspected lung lesions were evaluated by a multidisciplinary team and offered a range of investigative options, including CTGB if deemed clinically appropriate. Only patients undergoing CTGB were included in this study. Peripheral lung masses, which were primarily biopsied under ultrasound guidance by respiratory physicians,^7^ were excluded.

Before the procedure, patients were assessed in the interventional suite, and continuous monitoring was provided throughout the biopsy. Post-procedure, pulse and oxygen saturation were monitored continuously, while blood pressure and respiratory rate were recorded every 30 min for the first hour. A chest X-ray was performed 1 h after the procedure to assess for pneumothorax, with pneumothorax size categorised as small (<30%), moderate (30–50%) or large (>50%) using Collins’ method.^8^

### Discharge and conservative management criteria

Patients were stratified based on post-procedure chest X-ray findings. Those with no pneumothorax or a small, asymptomatic pneumothorax were discharged with verbal and written instructions to contact the pleural team (available 9am–5pm) or return to the emergency department outside these hours if they experienced worsening breathlessness, pain or general deterioration.

Patients with a moderate to large pneumothorax were observed for 4 h within the radiology recovery space, followed by a repeat chest X-ray. If they remained haemodynamically stable and met all of the following criteria, they were discharged without intervention and managed via the ambulatory pneumothorax pathway under pleural team supervision:

1. Oxygen saturation >90%
2. Respiratory rate <30 breaths per minute
3. Systolic blood pressure >90 mmHg
4. WHO performance status ≤2
5. Ability to self-care or availability of home support
6. Pain controlled with regular analgesic
7. No significant worsening of pneumothorax on repeat CXR.

Previously all patients with any pneumothorax post-CTGB were admitted to the hospital for observation and those with moderate/large pneumothorax had pleural aspiration or chest drain insertion.

The criteria for conservative management were adapted from Brown *et al* (2020),^9^ with pneumothorax size considered less critical than symptom severity and overall clinical stability when determining outpatient eligibility.

**Figure:**
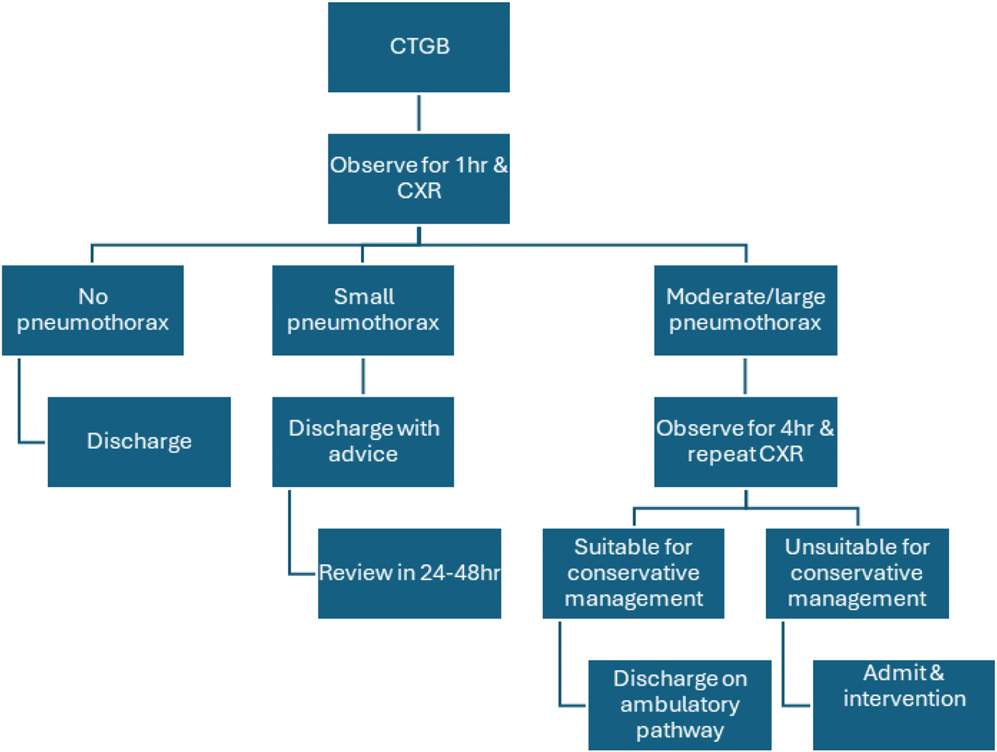
New pathway for CTGB

**Figure:**
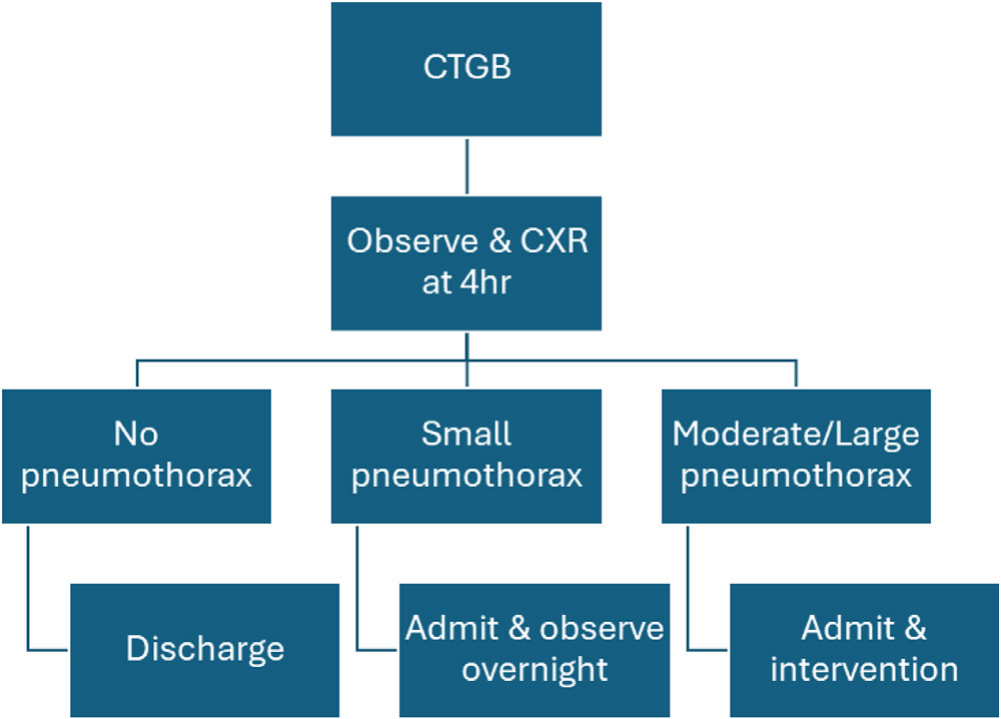
Old pathway for CTGB We have detailed our new and old CTGB pathways above in flowchart form.

### Follow-up and ambulatory care

Patients managed conservatively were followed up in the ambulatory care setting, with clinical review and repeat chest X-rays scheduled at 24–48 h, 1 week, 3 weeks and 7 weeks post-procedure or until pneumothorax resolution, whichever occurred first. To ensure close monitoring, the pleural team conducted a follow-up phone call the morning after discharge to assess symptoms and provide additional guidance. Patients and their caregivers were informed of red flag symptoms, such as uncontrolled pain, worsening breathlessness or general deterioration, and were advised to contact the pleural team during working hours or seek emergency care if needed.

To optimise hospital resource utilisation and reduce unnecessary visits, follow-ups were aligned with patients’ lung cancer clinic appointments whenever possible. During each visit, vital signs were reassessed, and a chest X-ray was performed to monitor pneumothorax progression. Pain management was optimised as needed, typically with co-codamol 30/500 mg (two tablets, four times daily) and Oramorph 5–10 mg as required every 4–6 h.

## Results

Between March 2021 and June 2023, a total of 213 consecutive patients were scheduled for CTGB. However, the procedure was ultimately performed in 207 patients, as five did not proceed due to resolving lesions, and one was unable to undergo biopsy due to the challenging position of the lesion.

Of the 207 consecutive patients who underwent CTGB, 42 (20.2%) developed iatrogenic pneumothorax. The mean age of the total group (207 patients) was 69 years (SD ± 8.09). Among them, 39 met the criteria for ambulatory management. 38 were managed on the ambulatory pathway, with one patient requiring admission following the procedure due to an episode of syncope. Three patients had a WHO performance status (PS) of three (two small and one large), two of whom required inpatient admission due to their inability to meet ambulatory care criteria. However, one patient with PS3, despite not meeting the standard criteria, was managed conservatively at their request, as they had strong home support and remained haemodynamically stable with a small pneumothorax.

Of the 38 patients managed conservatively on the ambulatory pneumothorax pathway (90.4% of those with pneumothorax), three (7.9%) ultimately required intervention. One patient required Thora-vent® (Uresil LLC) insertion after 3 weeks due to a slowly resolving pneumothorax, as thoracic surgeons sought rapid resolution before planned lung cancer surgery. Another required admission and chest drainage, as their family expressed inability to care for them as per our standard protocol, including conveyance for scheduled hospital visits. A third patient initially underwent Thora-vent® placement due to poorly controlled pain but later developed surgical emphysema, necessitating a chest drain. The biopsy procedure was successfully completed in 41 of the 42 patients who developed pneumothorax. In one case, the procedure was abandoned due to worsening breathlessness during the biopsy Table 1.

**Figure:**
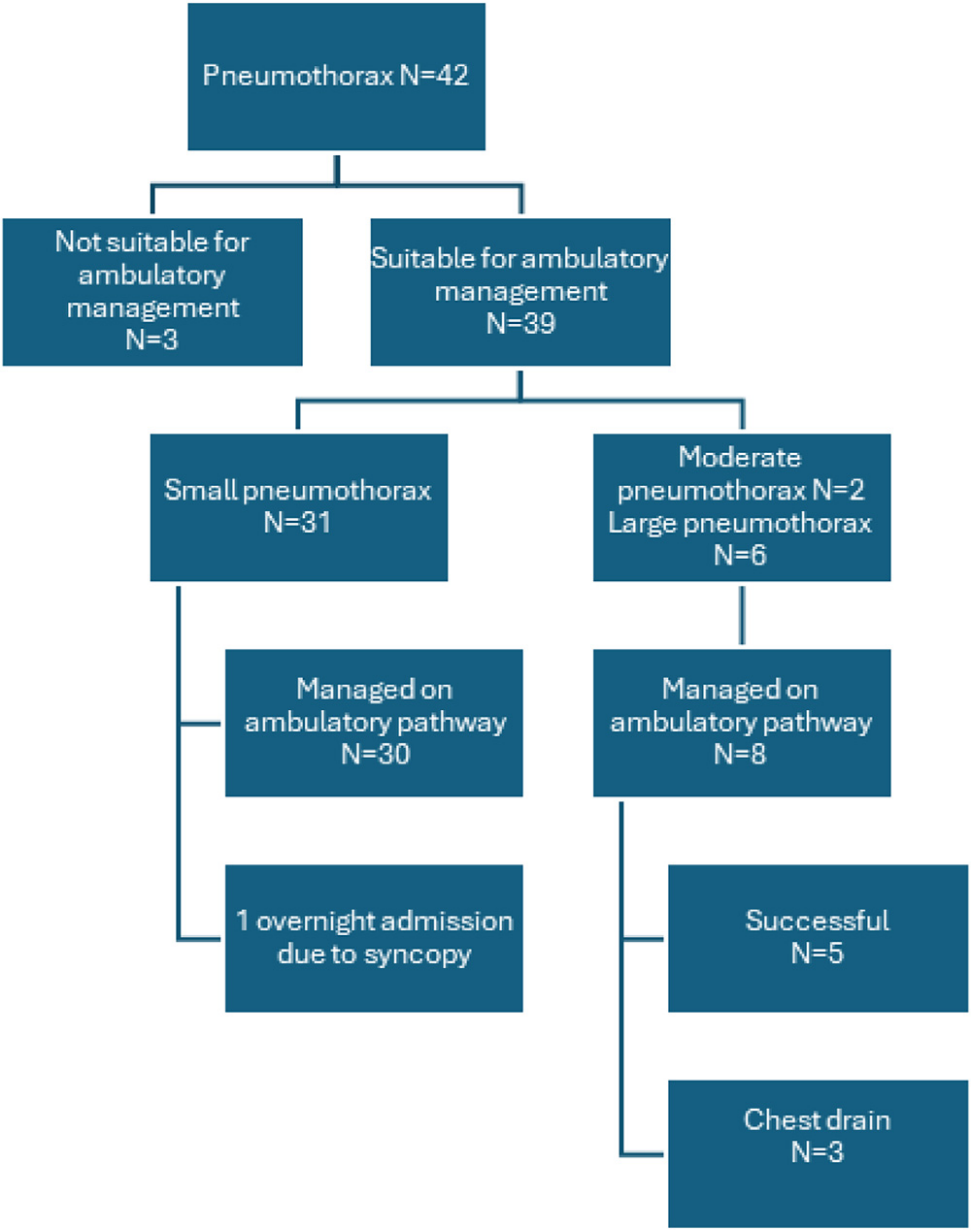
Flowchart of management of patients on ambulatory pathway

**Table 1:** Baseline characteristics.

Category	N
Total no of CTGB	213
Total no (%) of male patients	91 (42.72%) (91/213)
Total no (%) of female patients	122 (57.27%) (122/213)
No (%) of pneumothoraces	42 (20.28%) (42/207)
No (%) of male patients with pneumothorax	12 (28.57%) (12/42)
No (%) of female patients with pneumothorax	30 (71.42%) (30/42)
No (%) managed on ambulatory pathway	38 (90.47%) (38/42)
No (%) of patients requiring intervention	4 (9.52%) (4/42)

## Discussion

The concept of conservative management for pneumothorax is well-established. Stradling and Poole (1966) were among the earliest to advocate for a non-interventional approach in select cases of pneumothorax.^10^ More recently, Tavare *et al*^11^ demonstrated that most patients with iatrogenic pneumothorax following CTGB could be managed effectively on an ambulatory basis using a Heimlich valve chest drain, reducing the need for hospital admission. Similarly, Dennie *et al*,^12^ in a study of 506 patients undergoing transthoracic needle biopsy, found that 81% of those who developed iatrogenic pneumothorax remained asymptomatic and were safely discharged without immediate intervention. Only 1.4% of these patients later required further management, such as chest drains or pigtail catheters, due to the development of symptoms. Yamagami *et al*^13^ took a more interventional approach, performing manual aspiration of moderate-to-severe pneumothorax regardless of symptoms, suggesting that early aspiration may prevent progression. Despite the variability in management strategies for treatment of pneumothorax, a recent study by Brown *et al*^9^ raised the possibility of conservative management as a viable option. The study was, however, done on younger patients with primary spontaneous pneumothorax, as opposed to older patients with comorbidities undergoing CTGB.^9^

Our retrospective study of 213 patients reinforces this perspective, suggesting that CTGB can be safely performed as a short day-case procedure and that pneumothorax, when it occurs, can be effectively managed conservatively through ambulatory follow-up. In most cases, patients with small pneumothorax were discharged after 1 h of observation and those with moderate-to-large pneumothorax were observed for 4 h. Previously, these patients were admitted to hospital for overnight observation and/or intervention. The success of our approach was facilitated by a dedicated pleural team, which included specialist respiratory physicians and pleural nurses who provided close monitoring and timely intervention when required. This structured follow-up system allowed for a significant reduction in unnecessary hospital admissions and interventions.

A notable benefit of our ambulatory management pathway was its impact on procedural efficiency. Historically, our unit could accommodate only two procedures per session, as the available two spaces were entirely utilised for the 4 hr post-biopsy observation period. With the introduction of our new pathway, we were able to increase our capacity to three procedures per session, as most patients were discharged after an hour of procedure. This improvement reduced waiting times and expedited patient access to biopsy, enabling patients to undergo the procedure in the next available session without significant delays. Additionally, by eliminating the routine need for a booked recovery bed, we improved service flexibility and resource utilisation. There has been variability in practice with regards to the optimal observation time, with most centres suggesting at least 4 h of observation post-CTGB for all patients, followed by a repeat chest X-ray.^14^ In our practice, we were able to discharge all patients with no or small pneumothorax after 1 h of observation.

However, our study has limitations. As a single-centre, retrospective analysis conducted in a district general hospital (DGH), it is inherently prone to selection and institutional bias. The presence of a well-established pleural service and respiratory consultants with a special interest in pleural disease allowed for close monitoring and streamlined decision-making, which may not be easily replicable in all DGHs. Nonetheless, the simplicity of our ambulatory approach suggests that similar models could be implemented in other institutions with appropriate modifications, particularly through structured follow-up protocols and extended observation periods to ensure patient stability.

## Conclusion

This study demonstrates that most patients who develop iatrogenic pneumothorax following CTGB can be safely managed through conservative ambulatory care, with a low incidence of subsequent intervention. The integration of an outpatient CTGB service with a conservative pneumothorax management pathway significantly enhanced procedural capacity while ensuring safe and effective patient care. Notably, no serious complications were observed. Future research should evaluate the feasibility of implementing this model across diverse hospital settings to determine its broader applicability and potential for improving healthcare resource utilisation.

## Supporting information

The information leaflet produced by the respiratory and pleural team at Kettering General hospital. PI 1085 Pneumothorax – permission obtained.

## Funding

This research did not receive any specific grant from funding agencies in the public, commercial or not-for-profit sectors.

## Data availability statement

The data that support the findings of this study are available from the corresponding author upon reasonable request.

## Ethics approval and consent to participate

This study was a retrospective audit of the service and did not require formal ethics approval from the hospital. No identifiable or individualised patient data were used. All data were fully anonymised, and individual patient consent was not required.

## CRediT authorship contribution statement

**Syed Hasan Mustafa Rizvi:** Writing – review & editing, Writing – original draft, Resources, Project administration, Methodology, Investigation, Formal analysis, Data curation, Conceptualization. **Avik Banerjee:** Resources, Conceptualization. **Georgios Tsaknis:** Writing – review & editing, Supervision, Conceptualization. **Muhammad Naeem:** Project administration, Investigation. **Syed Mehdi:** Formal analysis, Data curation. **Samantha Rawson:** Data curation. **Raja Reddy:** Writing – review & editing, Supervision, Resources, Project administration, Methodology, Investigation, Conceptualization.

## Declaration of competing interest

The authors declare that they have no known competing financial interests or personal relationships that could have appeared to influence the work reported in this paper.

## References

[1] Zeng L., Liao H., Ren F. (2021). Pneumothorax induced by computedtomographyguided transthoracic needle biopsy: a review for the clinician. Int J Gen Med.

[2] Anzidei M., Porfiri A., Andrani F. (2017). Image-guided chest biopsies: techniques and clinical results. Insights Imaging.

[3] Wiener R.S., Wiener D.C., Gould M.K. (2013). Risks of transthoracic needle biopsy: how high?. Clin Pulm Med.

[4] Manhire A., Chairman M., Clelland C. (2003). Guidelines for radiologically guided lung biopsy. Thorax.

[5] NHS England (2023) NHS pressure continues as hospitals deal with high bed occupancy*.* https://www.england.nhs.uk/2023/01/nhs-pressure-continues-as-hospitals-deal-with-high-bed-occupancy/. Accessed January 1, 2025.

[6] Ewbank,L Thompson,J McKenna,H et al., (2021) NHS hospital bed numbers: past, present future*.* https://www.kingsfund.org.uk/publications/nhs-hospital-bed-numbers. Accessed January 21, 2025.

[7] Rizvi SHMR, Mirza M.M., Chotalia C.R. (2023). Physician-led US guided biopsy. Lung Cancer.

[8] Collins C.D., Lopez A., Mathie A., Wood V., Jackson J.E., ME Roddie (1995). Quantification of pneumothorax size on chest radiographs using interpleural distances: regression analysis based on volume measurements from helical CT. Am J Roentgenol.

[9] Brown S.G.A., Ball E.L., Perrin K. (2020). Conservative vs interventional treatment for spontaneous pneumothorax. N Engl J Med.

[10] Stradling P., Poole G. (1996). Conservative management of spontaneous pneumothorax. Thorax.

[11] Tavare A.N., Creer D.D., Khan S. (2016). Ambulatory percutaneous lung biopsy with early discharge and Heimlich valve management of iatrogenic pneumothorax: more for less. Thorax.

[12] Dennie C.J., Matzinger F.R., Marriner J.R., Maziak D.E. (2001). Transthoracic needle biopsy of the lung: results of early discharge in 506 outpatients. Radiology.

[13] Yamagami T., Nakamura T., Lida S. (2002). Management of pneumothorax after percutaneous CT-guided lung biopsy. Chest.

[14] Anzidei M., Sacconi B., Fraioli F. (2015). Development of a prediction model and risk score for procedure-related complications in patients undergoing percutaneous computed tomography-guided lung biopsy. Eur J Cardiothorac Surg.

